# Changes of intestinal flora in patients with systemic lupus erythematosus in northeast China

**DOI:** 10.1371/journal.pone.0213063

**Published:** 2019-03-14

**Authors:** Feng Wei, Huafeng Xu, Changxin Yan, Chunli Rong, Bingyu Liu, Haizhou Zhou

**Affiliations:** 1 Department of Laboratory Diagnosis, the First Affiliated Hospital of Harbin Medical University, Nangang District, Harbin, Heilongjiang, China; 2 Department of Radio-immunity, Heilongjiang Provincial Hospital, Harbin, Heilongjiang, China; Peking University First Hospital, CHINA

## Abstract

**Objective:**

The human gut harbors diverse microbes that play a fundamental role in the well-being of their hosts. Microbes can cause autoimmunity, trigger autoimmunity in genetically susceptible individuals or prevent autoimmunity. There were reports about intestinal flora changes in Systemic Lupus Erythematosus (SLE) patients, but no data were available in northeast China. In this study, we investigated the intestinal flora changes of SLE patients in Heilongjiang province located in northeast China.

**Methods:**

Feces from 16 SLE patients and 14 healthy volunteers were employed to extract bacterial DNA, amplify 16s RNA of bacteria, and analyze the biological information by sequencing. The statistical analysis used the SPSS version of 17.

**Result:**

We found that there were 1 phylums, 4 families and 9 genera in the intestinal flora of SLE patients. And the nine differences genera can be used to distinguish SLE patients from normal people.

**Conclusion:**

We found an increase of Proteobacteria and a decrease of Ruminococcaceae in SLE patients in different regions. In addition, we found that some proteins, enzymes, and diseases were significantly associated with SLE.

## Introduction

The human gut harbors diverse microbes that play a fundamental role in the well-being of their hosts [1]. Microbes can cause autoimmunity, trigger autoimmunity in genetically susceptible individuals or prevent autoimmunity [2]. Intestinal flora plays a very important role in the study of diseases and has been reported in autoimmune diseases [3]. In the past decades, a growing body of evidence has indicated an important role of gut microbes in the development of autoimmune diseases, including type 1 diabetes, rheumatoid arthritis, and multiple sclerosis [3–6]

Systemic lupus erythematosus (SLE) is a female multiple systemic autoimmune disease involving multiple organs throughout the body[7]. In recent years, there has been a lot of reports on the relationship between SLE and intestinal flora [7–9]. In Spain, the intestinal flora of SLE patients was characterized by a significantly lower Firmicutes/Bacteroidetes ratio and showed a depletion of *Lachnospiraceae* and *Ruminococcaceae* and an enrichment of *Bacteroidaceae* and *Prevotellaceae* [8]. In southern China, the intestinal flora of SLE patients was characterized by a significantly increase in Actinobacteria [9]. And at the family level, the *Ruminococcaceae* and *Desulfovibrionaceae* were significantly reduced, and the *Enterococcaceae* and *Bacteroidales_S24-7_group* were significantly increased [9]. In another study, the SLE patients lived in southern of China showed the depletion of Firmicutes, enrichment of Bacteroidetes, Actinobacteria and Proteobacteria, and signifcant increases of the family *Prevotellaceae*[7]. It can be seen that SLE patients have different intestinal flora changes in different regions. In northeast China, there is a unique geographical environment and diet culture, however, there was a lack of research on the changes of intestinal flora in patients with SLE.

In this study, we investigated the changes of intestinal flora in SLE patients in Heilongjiang province located in northeast China.

## Materials and methods

### 1. Ethical statement

Written informed consent was obtained in the local language from all participants. The First Affiliated Hospital of Harbin Medical University Ethics Review Committee granted ethical approval for the study.

### 2. Data collection

We performed a collection of fecal samples from patients diagnosed with SLE from the First Affiliated Hospital of Harbin Medical University from April 2018 to October 2018. These patients were diagnosed by clinicians as SLE at least a year ago according to the criteria set by the American College of Rheumatology (ACR). Patients had no other autoimmune diseases, as well as intestinal and metabolic diseases that affect the intestinal flora. The patients received no treatment for a month that affected the intestinal flora, including antibiotics, probiotics and other microbial preparations. Fourteen SLE patients were included in this study, and their clinical diagnosis and blood examination reports were obtained from the hospitals. Sixteen healthy volunteers were recruited by a routine physical examination. The healthy controls had no gastrointestinal tract disorders and did not receive any antibiotics within 1 month of this study. In addition, there were no significant differences among the two groups in terms of age, smoking history, and alcohol or dietary intake. All subjects included in this study provided written informed consents, and the protocol of this study was approved by the First Affiliated Hospital of Harbin Medical University.

### 3. DNA extraction from fecal specimens

Total DNA was extracted from thawed fecal samples using the QIAamp Fast DNA Stool Mini Kit (Qiagen, Hilden, Germany) according to the manufacturer protocols. The V3–V4 regions of the bacterial 16S rRNA gene sequences were amplifed from the diluted DNA extracts with the primers 338F (5′-ACTCCTACGGGAGGCAGCAG-3′) and 806R (5′-GGACTACHVGGGTWTCTAAT-3′).

### 4. PCR amplifcation and sequencing

PCR amplifcation was performed in a 20μl mixture containing 4μl of 5×FastPfu Buffer, 2μl of 2.5 mM dNTPs, 5×FastPfu Buffer, 0.8μl of forward primer (5μM), 0.8μl of reverse primer (5μM), 10ng of DNA sample, 0.2μl of BSA, 0.4μl of FastPfu Polymerase and added ddH2O to 20μl. The reactions were hot-started at 95°C for 3min, followed by 27 cycles of 95°C for 30 s, 55°C for 30 s, and 72°C for 45 s, with a final extension step at 72°C for 10min. Target PCR products (3μl) were visualized by electrophoresis in 2% agarose gel. After PCR products were purified and quantified, the pool of samples was mixed equimolar. Parallel tagged sequencing was performed using a Miseq Sequencing in Majorbio. The sequences sharing 97% similarity were grouped into the same operational taxonomic units (OTUs) using the MOTHUR program. Rarefaction analysis was carried out by Mothur and plot-rarefaction (Majorbio).

### 5. Biological information analysis: Species diversity and difference analysis

For comparing the OTUs, microbial diversity and richness of the three groups, the Sobs, Ace, Chao, PD, Heip, Shannon and Simpson indices were calculated through the rarefaction curves. Each sample was mapped based on the overall microbial composition and assessed for similarities.

To visualize the significantly altered genera, receiver operating characteristic (ROC) curves were plotted in the statistical programming language R (V.3.1.2). ROC curves are an effective method for evaluating the quality or performance of diagnostic tests and are widely used in gut microbiomes to evaluate the performance of many microbial biomarkers. Area under the curves (AUCs) of ROC was calculated to evaluate the performance of the fitted logistic regression models. The AUCs were based on the predicted probability of SLE for each individual, using the multivariate logistic regression coefficient estimates together with the individual’s transformed relative abundances for each bacterial taxon included in the analysis.

### 6. Function prediction analysis

We also used Phylogenetic Investigation of Communities by Reconstruction of Unobserved States (PICRUSt) to infer the functional content. The OTU abundance table is standardized by PICRUSt, and the Clusters of Orthologous Groups (COG) of proteins family information and KEGG Ortholog (KO) information corresponding to OTU were obtained through the greengene ids corresponding to each OTU, then the abundance and KO abundance of each COG were calculated. We compared COG abundance and KO abundance in SLE patients and healthy controls to predict the changes of intestinal flora functions in SLE patients.

### 7. Statistical analysis

SPSS version 17.0 was used for all statistical analysis. Single-sample t-test was used to compare the blood index of SLE patients with population average. Microbial phylum family and genera concentrations between SLE patients and healthy controls were compared using a Welch T test.

## Results

### 1. Clinical features of SLE patients

Clinical data including sex, age, disease duration, hemoglobin(Hb) concentrations, erythrocyte sedimentation rate(ESR), complement C3 concentrations and complement C4 concentrations are shown in Table 1. Compared with healthy controls, the ESR was significantly higher in patients with SLE (p<0.01). And, the Hb, C3 and C4 concentrations were significantly lower in SLE patients (p<0.05).

**Table 1:** Demographic and clinical characteristics of human subjects.

Characteristics	SLE patients	Healthy controls	*p* value
Sample numbers (female+male)	14 (13+1)	16 (14+2)	
Age mean±SD	40.71±13.85	38.63±14.50	>0.05
Hb(g/L) mean ±SD	105.73±34.84	132±9.69	0.014
ESR(mm/h) mean±SD	63.71±30.19	10±5.10	<0.0001
C3(g/L) mean±SD	0.71±0.23	1.35±0.05	<0.0001
C4(g/L) mean±SD	0.15±0.10	0.25±0.08	0.005
Disease duration (years) ± SD	4.86±5.23		

### 2. Structure and composition of the gut microbiome of SLE patients

**Fig 1:**
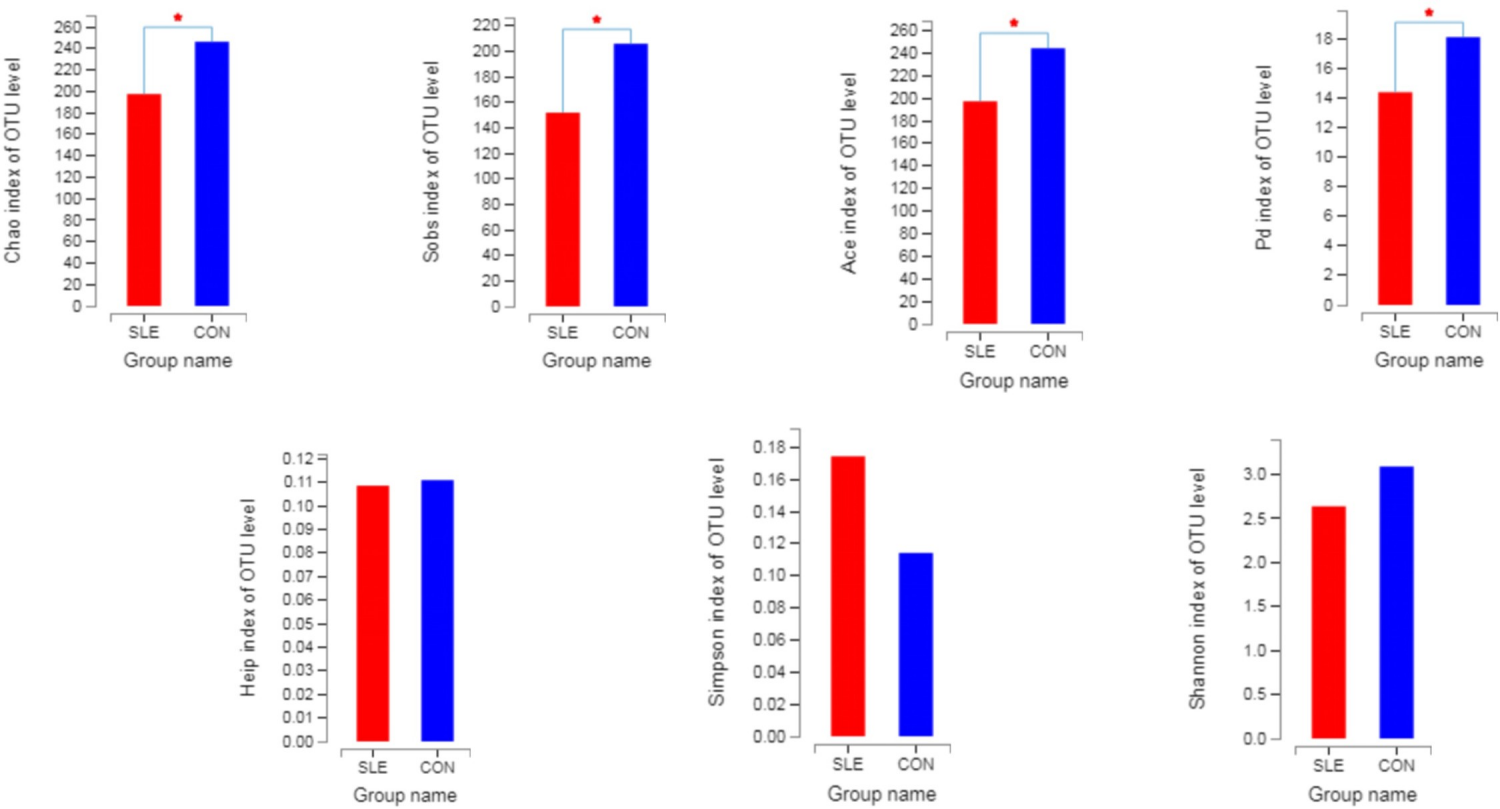
The alpha-diversity indices between SLE patients (red) and healthy controls (blue).

By detecting the bacterial sequences of feces from 14 SLE patients and 16 healthy controls, we obtained 1541337 sequences, the number of bases was 676677465 bp and the sequence average length was 439.4906542661 bp, and the number of phylum was 14, of family was 75, of genus was 225, of OUT was 663. The OUTs in both SLE patients and healthy controls were 466. About 85.98% of all OUTs was in SLE patients and about 79.39% of all OUTs was in healthy controls. As shown in Fig 1, not all of the alpha-diversity indices were different. Sobs, Chao, Ace and Pd index were signifcantly higher in healthy controls than in SLE patients, but there was no difference in Shannon, Simpson and Heip index in both groups. This indicated that the community richness and phylogenetic diversity of intestinal flora in patients with SLE were higher than those in normal controls, and there was no difference in community diversity and evenness between the two groups.

### 3. Microbiota changes of SLE patients

**Fig 2:**
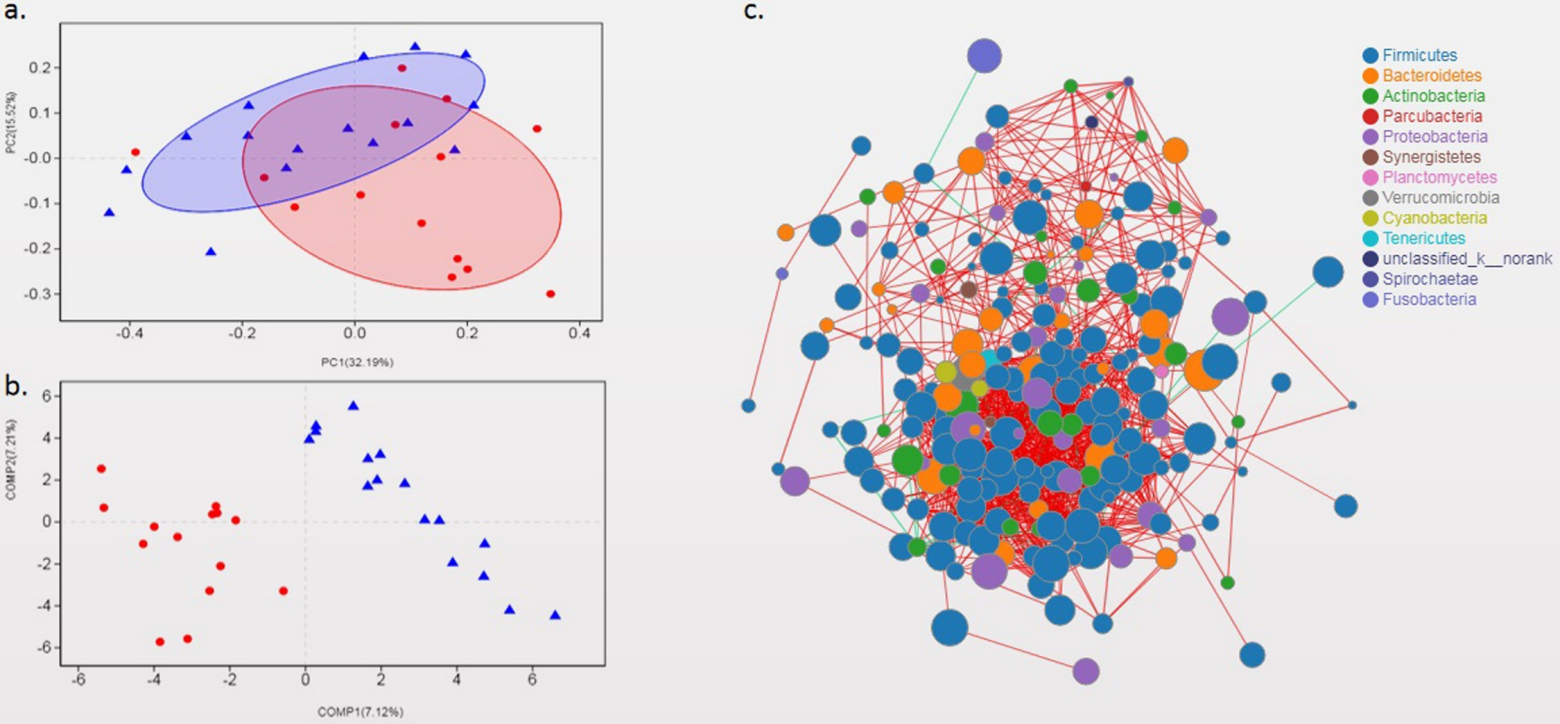
Gut microbial composition of SLE patients and healthy controls. (a) PCoA score plots of SLE patients (red) and healthy controls (blue) based on the gut microbial composition. (b) PLS-DA score plots of SLE patients(red) and healthy controls (blue) based on the gut microbial composition. (c) Species correlation network analysis on genus level. (The green line represents positive correlation, the red line represents negative correlation, the node represents species and the node size represents species abundance.)

**Fig 3:**
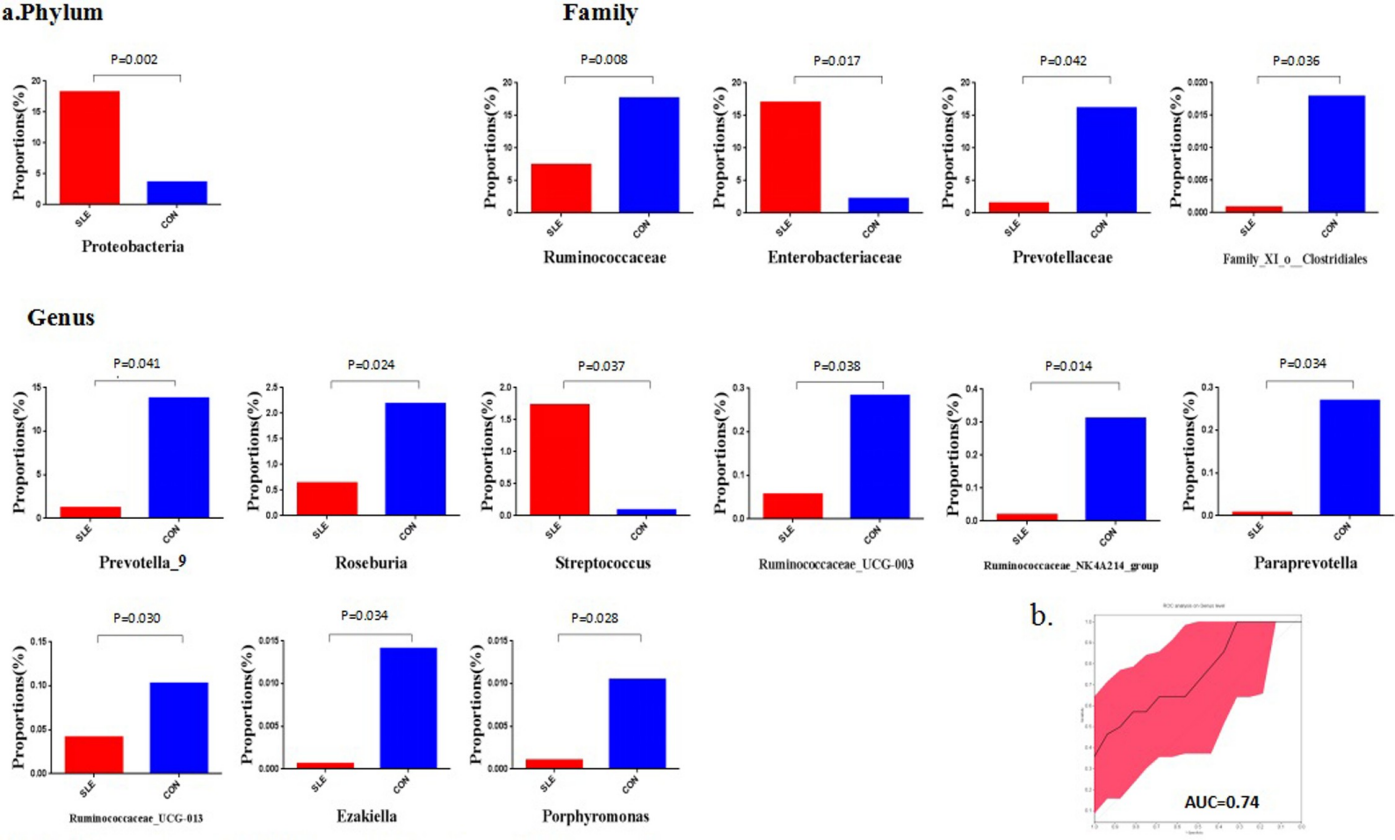
Differences in gut microbial between SLE patients and healthy controls. (a) Significantly altered gut microbiota between SLE patients (red) and healthy controls (blue) at the phylum, family and genus levels. (b) Receiver operating characteristic (ROC) curves demonstrating the performance of significantly altered microbial genera in SLE patients.

In genus level, the beta diversity difference using the weighted UniFrac distance was shown in PCoA analysis (Fig 2A) and the partial least squares discriminant analysis was shown in Fig 2B. The ordination plot demonstrated a clear difference between SLE patients and healthy controls. The species correlation network graph indicated there were intricate relationships between the genera (Fig 2C).There were not only intricate relationships between these bacteria, but also differences between SLE patients and normal controls (Fig 3A).

At the phylum level, the gut microbiota was dominated by Firmicutes, Bacteroidetes and Proteobacteria, which contributed to 90% of the gut microbiota, with smaller contributions of Actinobacteria and Fusobacteria. Compare to healthy controls, the signifcantly increased relative abundances of phylum in SLE patients was Proteobacteria (p = 0.002).

At the family level, compared to healthy controls, SLE patients had significantly increased relative abundances of the family *Enterobacterlaceae* (p = 0.017) and a decreased relative abundance of the family *Ruminococcaceae*(p = 0.008), family *Prevotellaceae* (p = 0.042) and *family_XI_o_Clostridiales* (p = 0.036).

At the genus level, compared to healthy controls, SLE patients had significantly increased relative abundances of the genus Streptococcus(p = 0.037) and a decreased relative abundance of the genus Prevotella_9(p = 0.041), Roseburia(p = 0.024), Ruminococcaceae_UCG-003(p = 0.038), Ruminococcaceae_NK4A214_group(p = 0.014), Paraprevotella(p = 0.034), Ruminococcaceae_UCG-013(p = 0.030), Ezakiella (E.za.ki.el'la N.L. fem. dim. Ezakiella named after the Japanese microbiologist Takayuki Ezaki who has contributed immensely to the taxonomy of the anaerobic Gram-stain positive cocci group of bacteria[10])(p = 0.034) and Porphyromonas(p = 0.028).

### 4. Evaluation of the performance of significantly changed genera

We conducted a ROC analysis to evaluate the utility of the differentially abundant genera as potential biomarkers. Nine significantly altered genera were screened for their abilities in distinguishing SLE patients fromhealthy controls. The results were shown in Fig 3B. Combination of significantly altered genera has the ability to differentiate SLE patients from healthy controls with AUC as 0.74.

### 5. Analysis of intestinal microbial function of SLE patients

**Fig 4:**
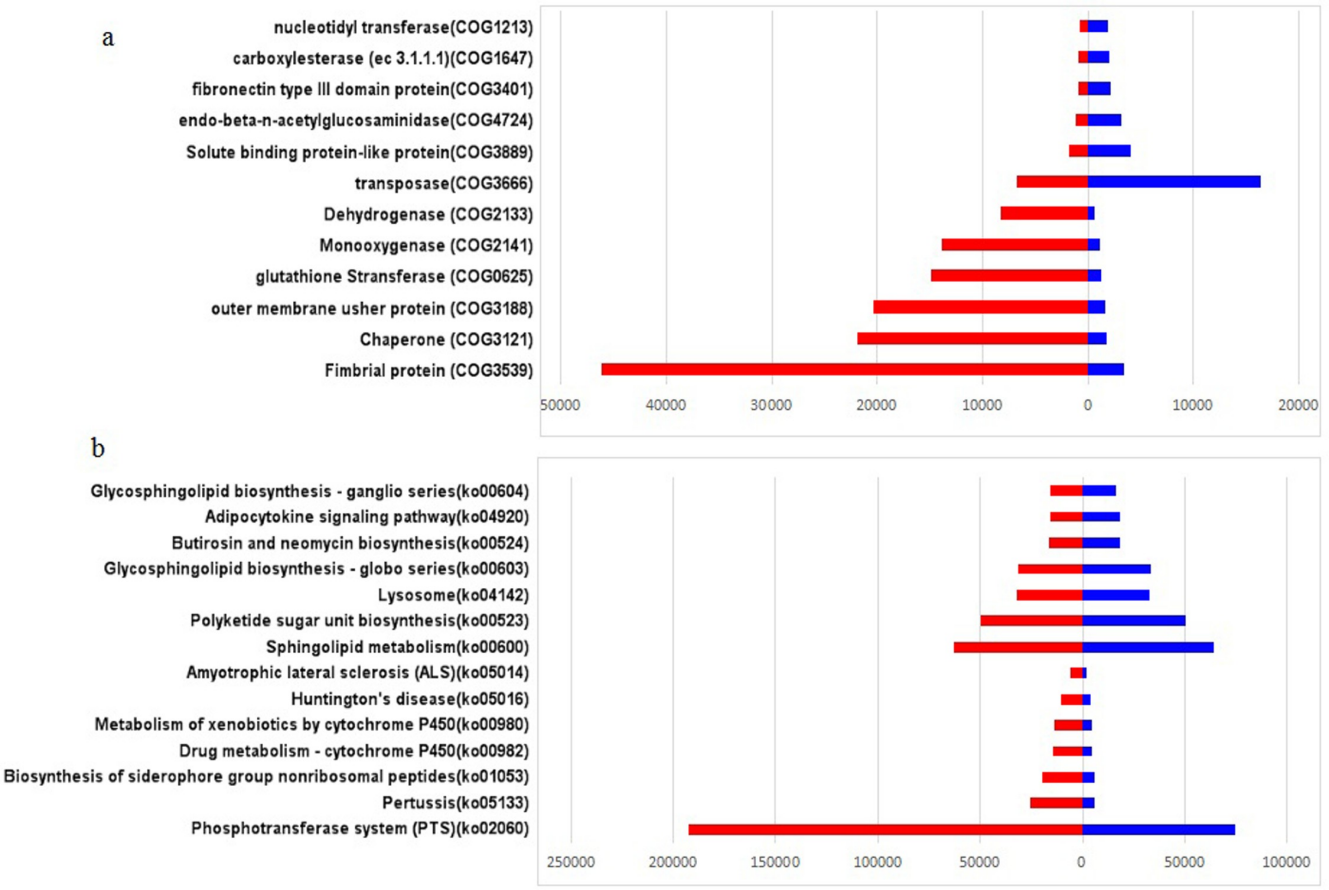
Analysis of intestinal microbial function of SLE patients. (a) Significantly alerted abundance of samples between SLE patients(red) and healthy control (blue) based on GOC data. (b) Significantly alerted pathway abundance of samples between SLE patients(red) and healthy control (blue) based on KEGG data.

To characterize the functional alterations of the gut microbiome in PBC, we predicted the functional composition profiles from 16S rRNA sequencing data with PICRUSt in SLE patients and healthy controls. We found that the abundance of COG samples in SLE patients was significantly different from those in healthy controls (Fig 4A). Compared with normal controls, the abundance of COG samples in SLE patients had significantly increased fimbrial protein (COG3539), chaperone (COG3121), outer membrane usher protein (COG3188), glutathione stransferase (COG0625), monooxygenase (COG2141) and dehydrogenase (COG2133), but decreased transposase(COG3666), solute binding protein-like protein(COG3889), endo-beta-n-acetylglucosaminidase (COG4724), fibronectin type III domain protein(COG3401), carboxylesterase (ec 3.1.1.1) (COG1647) and nucleotidyl transferase (COG1213). However, the abundance of KEGG pathway samples in SLE patients was significantly higher than that in healthy controls (Fig 4B), they were phosphotransferase system (PTS) (ko02060), pertussis(ko05133), biosynthesis of siderophore group nonribosomal peptides(ko01053), drug metabolism—cytochrome P450(ko00982), metabolism of xenobiotics by cytochrome P450(ko00980), huntington's disease(ko05016) and amyotrophic lateral sclerosis (ALS)(ko05014). Only seven KEGG pathway samples were significantly reduced in SLE patients, there were Sphingolipid metabolism(ko00600), Polyketide sugar unit biosynthesis(ko00523), Lysosome(ko04142), Glycosphingolipid biosynthesis—globo series(ko00603), Butirosin and neomycin biosynthesis(ko00524), Adipocytokine signaling pathway(ko04920) and Glycosphingolipid biosynthesis—ganglio series(ko00604).

## Discussion

It is well known that the intestinal flora is closely related to the living habits and diet of the population. The intestinal flora of SLE patients varies greatly due to their different regions of life. In this study, we found that the intestinal flora of patients with SLE in Heilongjiang Province, northenesat China, was different from that the SLE patients of foreign countries and southern China reported previously [7–9]. For example, at the phylum, we only found the phylum Proteobacteria was increased in SLE patients, this was different from SLE patients in Spain and southern China[7–9]. In Spain, the intestinal flora of SLE patients was characterized by a significantly lower Firmicutes/Bacteroidetes ratio and showed a depletion of *Lachnospiraceae* and *Ruminococcaceae* and an enrichment of *Bacteroidaceae* and *Prevotellaceae* [8]. In southern China, the intestinal flora of SLE patients was characterized by a significantly increase in Actinobacteria[9]. At the same time, at the family level, our study shown the significantly increased relative abundances of the family *Enterobacterlaceae* and a decreased relative abundance of the *family_XI_o_Clostridiales*, which was not changed in SLE patients of Spain and southern China [7–9].It is worth pointing out, in our study, the relative abundances of the family *Prevotellaceae* was significantly reduced, however, there was a significant increase in this family in SLE patients of Spain and southern China [7,9]. The difference of intestinal flora in different regions was also found in normal controls. The differences between the results of this study and others is possibly due in part to the unique geographical location and diatery habits. Therefore, this study is of great significance to improve the study of intestinal flora of SLE patients.

There are also plenty of studies showing similar changes in the intestinal flora of SLE patients with different cultures and diets.:

In phylum level, both the study of Zhixing He al. and ours revealed that an increase of Proteobacteria in SLE patients, compared to healthy controls [11]. In family level, our study indicated that a depletion of *Ruminococcaceae* in SLE patients, which is, similar to that of SLE patients of Spain[8] and of Zhejiang province in China[9],. The increase in Proteobacteria and the descrease in *Ruminococcaceae* was also associated with lupus nephritis with gastrointestinal damage [12]. Na-Ri Shin al. proposed that an increased prevalence of the bacterialphylum Proteobacteria is a marker for an unstable microbial community (dysbiosis) and a potential diagnostic criterion for disease [13]. Qingsen Shang al. found the *Ruminococcaceae* has been well illustrated to be responsible for the degradation of diverse polysaccharides and fibres[14]. Therefore, the increase of Proteobacteria and the decrease of *Ruminococcaceae* in SLE patients might indicate that these two microbes play an important role in the occurrence and development of SLE disease, but the specific mechanism needs to be further studied.

In addition to the above comparison of microflora in different populations, we also draw the following conclusions through functional prediction analysis.

First, compare to healthy control, some proteins were significantly increased in SLE patients, such as fimbrial protein, chaperone, outer membrane usher protein and siderophore group nonribosomal peptides. The increase of these proteins may be related to hypercoagulability [15], kidney injury [16], drug resistance [17] and control of some pathogenic microorganisms [18] in SLE patients. Secondly, the oxidation-related enzymes (such as monooxygenase, dehydrogenase and xenobiotics by cytochrome P450) and transferases(such as glutathione stransferase and PTS) were significantly increased in SLE patients. The increase of these enzymes may be related to oxidative stress [19,20], signal pathway protein phosphorylation[21] and drug metabolism [22] in patients with SLE. Thirdly, the huntington's disease and ALS are also associated with SLE. Huntington's disease and ALS are both neurodegenerative diseases [23]. Some studies have shown that neurodegenerative diseases are related to intestinal flora [24,25], so we speculated that SLE had this correlation with the changes of intestinal flora of these two neurodegenerative diseases.

However, only seven groups of metabolic pathways were significantly reduced in SLE patients, including Sphingolipid metabolism, Polyketide sugar unit biosynthesis, Lysosome, Glycosphingolipid biosynthesis—globo series, Butirosin and neomycin biosynthesis, Adipocytokine signaling pathway and Glycosphingolipid biosynthesis—ganglio series. But the correlation between these functions and the flora needs further study.

## Conclusion

All in all, we found an increase of Proteobacteria and a decrease of Ruminococcaceae in SLE patients in different regions. And nine genera were found to distinguish SLE patients from normal controls. Also, we found that some proteins, enzymes, and diseases were significantly associated with SLE. These result indicated the phylum Proteobacteria and the family Ruminococcaceae may be closely related to the occurrence and development of SLE, which may provide a new direction for the study of SLE in the future. And nine mutant genera can distinguish between SLE and normal people. And we found that some of the differences in proteins and enzymes between SLE patients and normal people may be due to differences in flora, so we can use the way of metabolomics and macrogenomics to further study the correlation between microflora and changes in these substances. At the same time, we found that there is a link between SLE and Huntington's disease and ALS, which can provide a new way to study the three diseases and thus find the link between them.

## Supporting information

S1 Table Data of alpha-diversity indices between SLE patients and healthy controls. (XLS)

S2 Table Data of PCoA of SLE patients and healthy controls. (XLS)

S3 Table Data of PLS-DA of SLE patients and healthy controls. (XLS)

S4 Table Data of species correlation network analysis of SLE patients and healthy controls. (XLS)

S5 Table Data of differences in gut microbial between SLE patients and healthy controls. (XLS)

S6 Table Receiver operating characteristic (ROC) data of significantly altered microbial genera in SLE patients. (XLS)

S7 Table GOC data and KEGG data of SLE patients. (XLS)
